# Mitochondrial tRNA^Gln^ 4394C>T Mutation May Contribute to the Clinical Expression of 1555A>G-Induced Deafness

**DOI:** 10.3390/genes13101794

**Published:** 2022-10-05

**Authors:** Yu Ding, Yaoshu Teng, Qinxian Guo, Jianhang Leng

**Affiliations:** 1 Central Laboratory, Hangzhou First People’s Hospital, Zhejiang University School of Medicine, Hangzhou 310006, China; 2 Department of Otolaryngology, Hangzhou First People’s Hospital, Affiliated to Zhejiang University School of Medicine, Huansha Road No. 261, Hangzhou 310006, China

**Keywords:** deafness, mt-tRNA^Gln^ 4394C>T mutation, m.1555A>G mutation, synergistic effect

## Abstract

The mitochondrial 1555A>G mutation plays a critical role in aminoglycoside-induced and non-syndromic hearing loss (AINSHL). Previous studies have suggested that mitochondrial secondary variants may modulate the clinical expression of m.1555A>G-induced deafness, but the molecular mechanism has remained largely undetermined. In this study, we investigated the contribution of a deafness-associated tRNA^Gln^ 4394C>T mutation to the clinical expression of the m.1555A>G mutation. Interestingly, a three-generation family with both the m.1555A>G and m.4394C>T mutations exhibited a higher penetrance of hearing loss than another family harboring only the m.1555A>G mutation. At the molecular level, the m.4394C>T mutation resides within a very conserved nucleotide of tRNA^Gln^, which forms a new base-pairing (7T-66A) and may affect tRNA structure and function. Using trans-mitochondrial cybrid cells derived from three subjects with both the m.1555A>G and m.4394C>T mutations, three patients with only the m.1555A>G mutation and three control subjects without these primary mutations, we observed that cells with both the m.1555A>G and m.4394C>T mutations exhibited more severely impaired mitochondrial functions than those with only the m.1555A>G mutation. Furthermore, a marked decrease in mitochondrial RNA transcripts and respiratory chain enzymes was observed in cells harboring both the m.1555A>G and m.4394C>T mutations. Thus, our data suggest that the m.4394C>T mutation may play a synergistic role in the m.1555A>G mutation, enhancing mitochondrial dysfunctions and contributing to a high penetrance of hearing loss in families with both mtDNA pathogenic mutations.

## 1. Introduction

Deafness is a common sensory defect, with an incidence of 2.8 in 1000 children [1]. This disease is related to single-gene mutations or environmental risk factors such as aminoglycoside antibiotics (AmAn). In fact, ~50% of AINSHL cases have a genetic etiology, and >150 nuclear genes, together with >1200 mutations, have been confirmed to be associated with hearing loss (http://deafnessvariationdatabase.org/ (accessed on 11 September 2022) [2,3]. Mitochondrial DNA (mtDNA) mutations have been thought to be associated with both syndromic and nonsyndromic hearing loss [4,5]. Among these, the m.1555A>G and m.1494C>T mutations in the extremely conserved A-site of 12S rRNA have been found to be associated with AINSHL in many families worldwide [6,7,8]. Without the use of AmAn, individuals with the m.1555A>G mutation exhibit a variable degree of hearing impairment, from profound to normal [9,10]. Furthermore, functional characterization of cell lines derived from subjects with the m.1555A>G mutation shows that it confers a mild impairment of mitochondrial function and is sensitive to AmAn [11,12], suggesting that this mutation is insufficient by itself to form enough phenotypes, and that therefore a combination of environmental factors, nuclear genes and mitochondrial genetic variants may contribute to deafness expression [13,14,15]. In particular, it has been documented that mt-tRNA^Ile^ 4317A>G, tRNA^Glu^ 14693A>G and tRNA^Cys^ 5802T>C variants may increase the penetrance and expressivity of the m.1555A>G mutation [16,17,18]. Nevertheless, the pathogenesis of mtDNA secondary variants remains unclear.

Previously, we established a new multiplex allele-specific PCR (MAS-PCR) that could screen for the presence of deafness-related m.1555A>G or m.1494C>T mutations. This MAS-PCR is a simple, inexpensive, fast and reliable method, which can be used to detect the deafness-associated 12S rRNA mutations in the general population [19]. To further assess its accuracy, we screened for the presence of the m.1555A>G and m.1494C>T mutations in a cohort of 500 deaf patients and 300 controls [20]. During that process, we identified a novel tRNA^Gln^ 4394C>T mutation, together with the m.1555A>G mutation, in a Chinese family with maternal transmission of AINSHL (ID: HZD510), which manifested a much higher penetrance of hearing impairment (50% including AmAn and 10% excluding AmAn) than another pedigree (ID: HZD055) with only the m.1555A>G mutation (30% including AmAn and 0% excluding AmAn) which harbored the same mitochondrial haplogroup D4g2a as HZD510 [21]. Intriguingly, the m.4394C>T mutation was located at conventional position 7 in the Acceptor arm of tRNA^Gln^, which was conserved from various vertebrates. Importantly, the m.4394C>T mutation formed a novel nucleotide pairing (7T-66A), while the m.4269A>G mutation, which occurred at the same position in the tRNA^Ile^ had been linked to cardiomyopathy [22,23]. Thus, we hypothesized that the m.4394C>T mutation, which may be similar to the m.4269A>G mutation, could also impair the mitochondrial function that is involved in deafness expression. To further understand the functional roles of the m.4394C>T mutation, trans-mitochondrial cells (cybrids) were generated in three patients from the HZD510 pedigree harboring both the m.1555A>G and m.4394C>T mutations, three patients from the HZD055 pedigree with only the m.1555A>G mutation and three healthy subjects without these mtDNA mutations. We found that the m.4394C>T mutation could enhance mitochondrial dysfunctions due to the m.1555A>G mutation.

## 2. Materials and Methods

### 2.1. Subjects and Audiological Assessments

**Figure 1:**
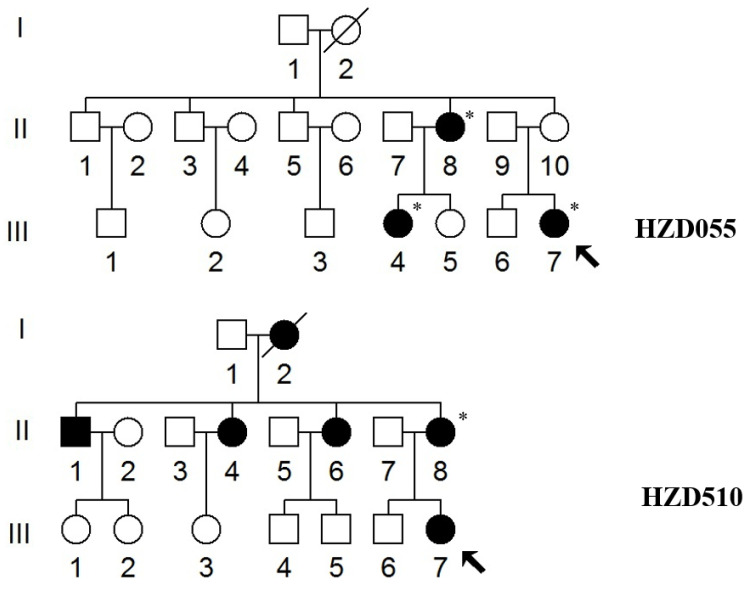
Two Han Chinese pedigrees with AINSHL. Arrows indicate the probands, * indicates the individuals who had a history of using AmAn.

Two Han Chinese families (HZD055 and HZD510) with hearing impairment were selected from Hangzhou First People’s Hospital (Figure 1). The patients’ demographics and family medical history were gathered, and blood samples were collected in our laboratory. Our study was approved by the Ethics Committee of Hangzhou First People’s Hospital (No: 2020-285-01), each subject providing his/her written informed consent. Furthermore, the consent for publication of their cases was also obtained from each individual. In addition, 300 healthy subjects (169 males and 131 females), aged from 19 to 55, with an average age of 39 years, were enrolled as controls.

In addition, pure-tone audiometry (PTA) was carried out in a sound-controlled room at frequencies ranging from 250 to 8000 Hz, as suggested in our recent study [24]. The levels of hearing loss were divided into five grades: normal: *<*20 decibels (dB); mild: 20–40 dB; moderate: 41–70 dB; severe: 71–95 dB; and profound *>*95 dB, according to the study as previously described [25].

### 2.2. mtDNA Analysis

The genomic DNA of the affected matrilineal relatives of these pedigrees was isolated using Puregene DNA isolation kits (Biomega). 24 PCR primers spanning complete mtDNA genes were used to amplify the mitochondrial genomes, as suggested in a previous study [26]. The PCR products were purified and analyzed, and subsequently compared with an updated version of the human mitochondrial genome sequence to detect the mutations (GenBank Accessible No: NC_012920.1) [27,28]. 

### 2.3. Data Analyses

The ClustalW program (http://www.ebi.ac.uk/Tools/msa/clustalw2/ (accessed on 11 September 2022) was used to align the mtDNA sequences. A total of 14 species were selected for this analysis. The conservation index (CI) was measured by comparing the human mtDNA with that of other vertebrates. CI ≥ 75% was considered to have functional significance [29]. Moreover, the Phylotree (http://www.phylotree.org/ (accessed on 11 September 2022)) and East Asia phylogeny were used to determine the mtDNA haplogroups of these two pedigrees [21,30]. 

### 2.4. Screening for Nuclear Gene Mutations

To see the contributions of common nuclear modified genes (*GJB2*, *GJB3*, *GJB6*, *SLC26A4* and *TRMU*) to m.1555A>G-induced deafness, we conducted mutational screening for these genes in two pedigrees (HZD055 and HZD510). PCR-Sanger sequencing was performed using the method described previously [31,32].

### 2.5. Generation of Cell Lines and Culture Conditions

Nine trans-mitochondrial cells which derived from three patients with both the m.1555A>G and m.4394C>T mutations (HZD510: II-6, II-8 and III-7), three individuals with only the m.1555A>G mutation (HZD055: II-8, III-4 and III-7) and three controls without these mtDNA mutations (C1, C2 and C3). These were constructed by introducing mitochondria from immortalized lymphoblastoid cell lines into human ρ° cells, which were generated from bromodeoxyuridine (BrdU)-resistant 143B cells, according to the protocols described elsewhere [33]. All cells were cultured in RPMI1640 medium (Invitrogen), supplemented with 10% cosmic calf serum. 

### 2.6. ATP Analysis

The intracellular ATP levels in cell lines derived from the six deaf patients (HZD510: II-6, II-8 and III-7; HZD055: II-8, III-4 and III-7), together with the three controls (C1, C2 and C3), were determined using Cell Titer-Glo^®^ Luminescent Cell Viability Assay (Promega), according to the manufacturer’s protocols [34].

### 2.7. Analysis of Mitochondrial Membrane Potential (MMP)

The levels of MMP in nine cybrid cells were evaluated using JC-1 dye (Life Technology, Carlsbad, CA, USA). At a low concentration, JC-1 existed as a monomer and was detected as green fluorescence, while at a high concentration, JC-1 existed as a multimer and was detected as red fluorescence [35]. To measure the MMP, JC-1 staining buffer was first added to the cells for 20 min. Subsequently, the solution was removed, and the cells were washed with JC-1 twice. After adding PBS per well, the fluorescence signal of the cells was observed under flow cytometry (BD Biosciences, Franklin Lakes, NJ, USA) [36].

### 2.8. Mitochondrial Reactive Oxygen Species (ROS) Analysis

A total of 2 × 10^6^ cells were maintained in fluorescent probe 2′-7′dichlorofluorescin diacetate (DCFH-DA) with a final concentration of 10 μM at 37 °C for 0.5 h. Subsequently, the cells were washed twice with PBS and analyzed using a fluorescence plate reader (Millipore, Burlington, MA, USA) at 485 nm excitation and 535 nm emission [37].

### 2.9. mtDNA Quantification

Quantitative PCR (qPCR) was applied to quantify the mtDNA content. The mtDNA *ND1* was used to determine the mtDNA copy numbers, and the nuclear β-globin gene was used as a control. The primer sequence for the amplification of the mt-*ND1* was: forward: 5′-AACATACCCATGGCCAACCT-3′; reverse: 5′-AGCGAAGGGTTGTAGTAGCCC-3′. The primer for the genetic amplification of the β-globin gene was: forward: 5′-GAAGAGCCAAGGACAGGTAC-3′; reverse: 5′-CAACTTCATCCACGTTCACC-3′. The qPCR data were normalized to β-globin, then compared with the wild-type control values and analyzed using a relative quantification method (2^−ΔΔCT^) [38].

### 2.10. Measurement of mt-RNA Transcription

The total RNA was isolated from nine cybrids using the TRIzol reagent (Thermo Fisher Scientific, Waltham, MA, USA). In brief, 500 ng of RNA was used with a reverse transcription kit (Takara, Kusatsu, Shiga, Japan). Then, fluorogenic SYBR Green (Bio-Rad, Hercules, CA, USA) was used for qPCR, following the protocol suggested previously [39]. The primers for the qPCR amplification are listed in Table 1.

### 2.11. Assays of Activities of Respiratory Chain Complexes

The enzymatic activities of complexes I, II, III and IV were assayed as detailed elsewhere [16], and their activities were normalized by citrate synthase activity.

### 2.12. Qualification of 8-Hydroxy-2′-Deoxyguanosine (8-OHdG)

The presence of 8-OHdG is commonly regarded as an indicator of oxidative stress and mitochondrial dysfunction [40]. The plasma concentrations of 8-OHdG in three subjects with both the m.1555A>G and m.4394C>T mutations, three subjects with only the m.1555A>G mutation and three controls were analyzed using enzyme-linked immunosorbent assays (ELISA), according to the protocol provided by the manufacturer (Nikken Foods, St. Louis, MO, USA).

### 2.13. Assigning Pathogenicity to the mt-tRNA^Gln^ Mutation

The updated pathogenicity scoring system was used to assess the m.4394C>T mutation [41]. The mutation was classified as “probably pathogenic” with a score of ≥11 points, “possibly pathogenic” with a score of 7–10 points and a “neutral polymorphism” with a score of ≤6 points.

### 2.14. Computer Analysis

All data were expressed as the mean ± standard deviation (SD). The statistical analyses were carried out using the unpaired, two-tailed Student’s *t* test contained in the GraphPad Prism 5 program (GraphPad Software). *p* < 0.05 was believed to be statistically significant. 

## 3. Results

### 3.1. Clinical Features of HZD055 and HZD510 Pedigrees

**Figure 2:**
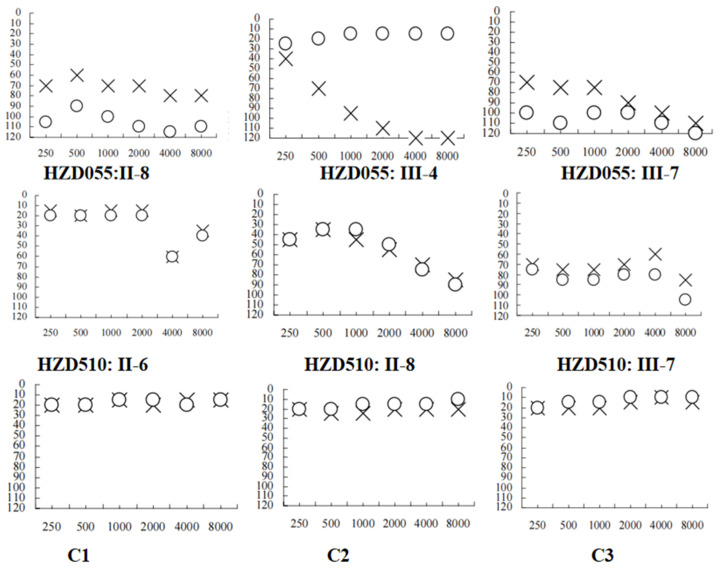
Audiograms of several matrilineal relatives in two Chinese families with AINSHL. X: left ear; O: right ear; C1: HZD055: II-6; C2: HZD055: III-1; C3: HZD055: III-3.

We examined two Chinese families with maternally transmitted AINSHL in the Otology Clinic of Hangzhou First People’s Hospital (Figure 1). In the HZD055 pedigree, the proband (III-7) lived in Hangzhou city of Zhejiang Province. She had a history of using gentamycin when she was 19 years old, and she began to suffer from bilateral hearing loss two months after she started using gentamycin. Clinical examinations revealed that she exhibited bilateral hearing loss (106 dB for the right ear, 86 dB for left ear). Interestingly, her matrilineal relatives (II-8 and III-4) also had a history of using AmAn. As shown in Figure 2, they had profound hearing impairment.

In the HZD510 pedigree, five of the ten matrilineal relatives exhibited deafness as a sole clinical phenotype. Genetic counseling suggested that the matrilineal relatives (II-1, II-4, II-6, II-8 and III-7) were deaf patients. Audiological examinations revealed that subject II-6 had mild hearing loss, subject II-8 had moderate hearing loss and proband (III-7) developed severe hearing impairment (Figure 2 and Table 2). A comprehensive genetic counseling revealed that these subjects did not have any other clinical abnormalities, such as cardiovascular disease, visual loss, trauma, infectious diseases or neurological disorders.

As shown in Figure 1, the family histories of HZD055 and HZD510 were consistent with maternal inheritance. Notably, the penetrance of hearing loss in HZD055 and HZD510 was 30% and 50% (AmAn included), respectively; when the AmAn was excluded, the penetrance of these pedigrees was 0% and 10%, respectively. The age at onset of deafness in HZD055 and HZD510 varied from 19 to 45, with an average of 27 years. 

### 3.2. Detecting the Deafness-Associated mtDNA Mutations

**Figure 3:**
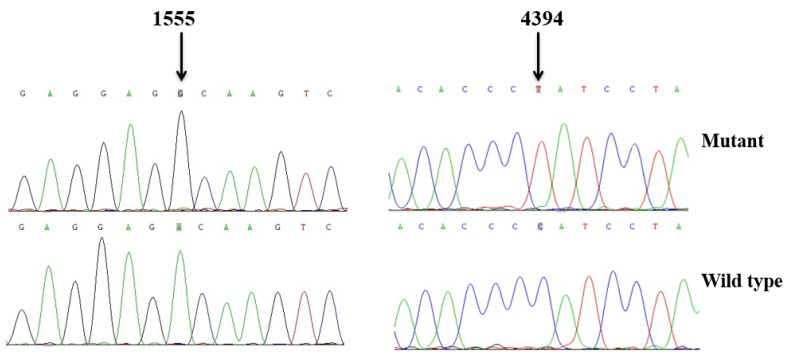
Identification of the m.1555A>G and m.4394C>T mutations via Sanger sequencing, partial sequence chromatograms of 12S rRNA and tRNA^Gln^ gene from affected individual and a Chinese control. Arrows indicate the location of the base changes at positions 1555 and 4394.

We used PCR and Sanger sequencing technologies to screen mtDNA mutations/variants in the matrilineal relatives of the HZD055 and HZD510 pedigrees. Compared with the rCRS, we identified a set of mtDNA mutations which belonged to East Asian mitochondrial haplogroup D4g2a [21]. As shown in Table 3, there were 14 variants in D-loop, 4 variants in 12S rRNA, 3 variants in 16S rRNA, 1 mutation in tRNA^Gln^ (4394C>T), as well as the 9-bp deletion in the nucleotide 8271–8279, whereas the rest were located at oxidative phosphorylation (OXPHOS)-related genes. In addition, ten missense mutations were found in this study, including *ND2* 4491G>A (p.V8I), 5178C>A (p.L237M), *COII* 7976G>A (p.G131S), *A8* 8414C>T (p.L17F), 8584G>A (p.A20T), *A6* 8701A>G (p.T59A), 8860A>G (p.T112A), *ND3* 10398A>G (p.T112A), *CytB* 14766C>T (p.T7I) and 15326A>G (p.T194A). These mtDNA variants were further assessed by evolutionary conservation analysis in four species: human [28], bovine [42], mouse [43] and *Xenopus laevis* [44]. We found that aside from the m.1555A>G and m.4394C>T mutations (Figure 3), other variants were not well conserved. In addition, the m.1555A>G and m.4394C>T mutations were not detected in the 300 controls, suggesting that they may be associated with AINSHL.

**Figure 4:**
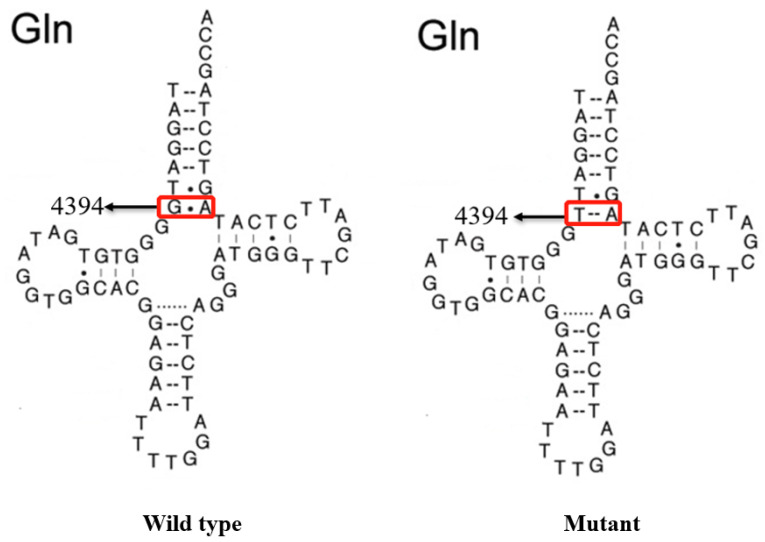
Secondary structure of mt-tRNA^Gln^ with and without the m.4394C>T mutation, which is derived from Mitomap database (www.mitomap.org). Arrows denoted the position of the m.4394C>T mutation.

**Figure 5:**
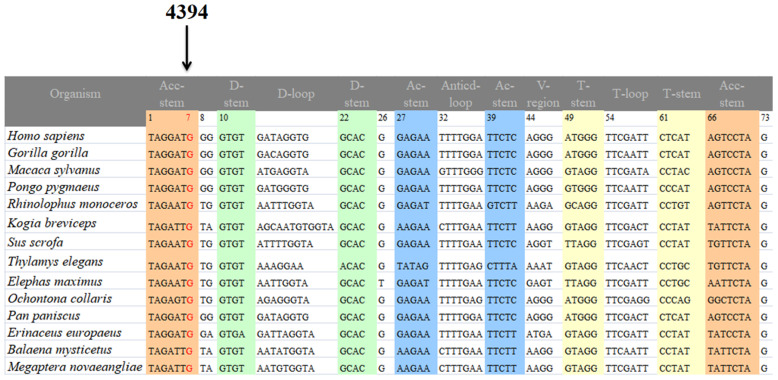
Sequence alignment of the mt-tRNA^Gln^ gene from 14 species. The arrow indicates the m.4394C>T mutation.

As shown in Figure 4 and Figure 5, the homoplasmic m.4394C>T mutation resided at position 7 in the Acceptor arm of tRNA^Gln^, which was extremely conserved from various species. Importantly, the m.4394C>T mutation formed a new 7T-66A base-pairing and may have a structural and functional impact on tRNA^Gln^.

### 3.3. Construction of Cybrid Cells from Two Chinese Families with AINSHL

**Figure 6:**
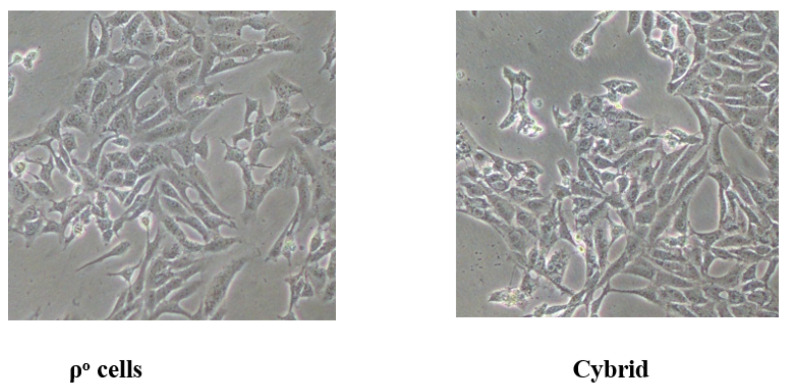
The cellular morphology of human ρ° and cybrids cells.

Immortalized lymphoblastoid cell lines were generated from three matrilineal relatives (HZD510: II-6, II-8 and III-7) harboring both the m.1555A>G and m.4394C>T mutations, three subjects (HZD055: II-8, III-4 and III-7) belonging to the mtDNA haplogroup D4g2a, and three controls without these pathogenic mutations (C1, C2 and C3) (Figure 6). The cybrid cells were used to further the biochemical analysis.

### 3.4. Decreased in ATP Production

**Figure 7:**
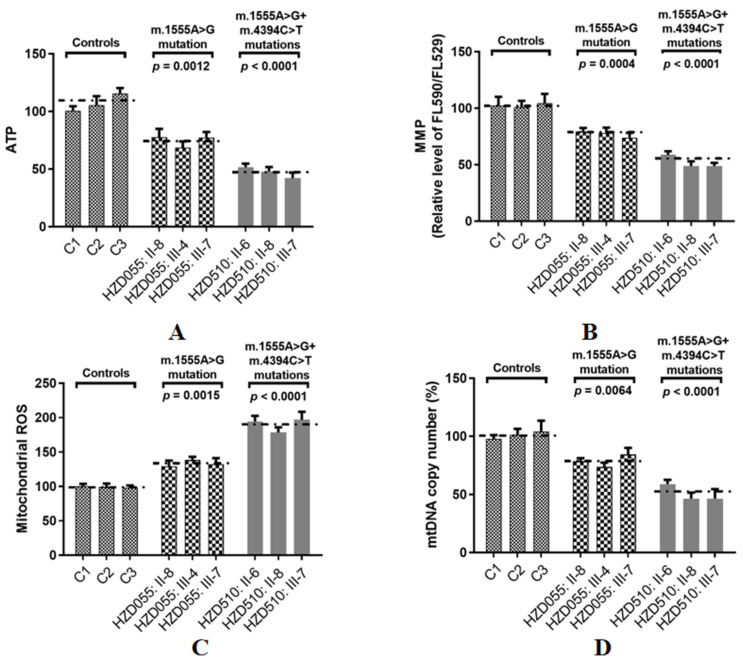
Assessments of mitochondrial functions in three patients with both the m.1555A>G and m.4394C>T mutations, three patients with only the m.1555A>G mutation and three controls. (**A**) Analysis of ATP production in nine cybrids; (**B**) MMP analysis in cybrids; (**C**) Qualification of ROS levels; (**D**) Determining the mtDNA content.

ATP is the key energy-carrying molecule in all cells. As shown in Figure 7A, the ATP levels in cell lines with both the m.1555A>G and m.4394C>T mutations and the cells with only the m.1555A>G mutation were 48% (*p* < 0.001) and 74% (*p* = 0.0012) of the average value measured in the control cell lines, respectively.

### 3.5. Reductions in MMP

Depolarization of MMP is often associated with apoptosis by the cytochrome c released from mitochondria [45]. To see whether the m.4394C>T mutation affected MMP, the JC-1 assay was used to evaluate the MMP in both mutant and control cells. As shown in Figure 7B, the levels of MMP in the mutant cell lines carrying both the m.1555A>G and m.4394C>T mutations and the cells carrying only the m.1555A>G mutation were 53% (*p* < 0.0001) and 74% (*p* = 0.0004) of the mean values measured in the control cell lines, respectively.

### 3.6. The Increase in ROS Production

ROS plays an active role in many cellular process [46]. As illustrated in Figure 7C, the levels of ROS generation in mutant cell lines with both the m.1555A>G and m.4394C>T mutations and the cells carrying only the m.1555A>G mutation were 192% (*p* < 0.0001) and 137% (*p* = 0.0015) of the mean values measured in the control cell lines, respectively.

### 3.7. mtDNA Copy Number Decrease

The mtDNA copy number is a promising biomarker of mitochondrial dysfunction [47]. To see whether the m.4394C>T mutation affected mtDNA content, Qpcr was used to assess the mtDNA copy number variations. As shown in Figure 7D, the levels of the mtDNA copy number in the mutant cell lines carrying both the m.1555A>G and m.4394C>T mutations and the cells carrying only the m.1555A>G mutation were 52% (*p* < 0.0001) and 78% (*p* = 0.0064) of the mean values measured in control group, respectively.

### 3.8. Effect of the m.4394C>T Mutation on mt-RNA Transcription

**Figure 8:**
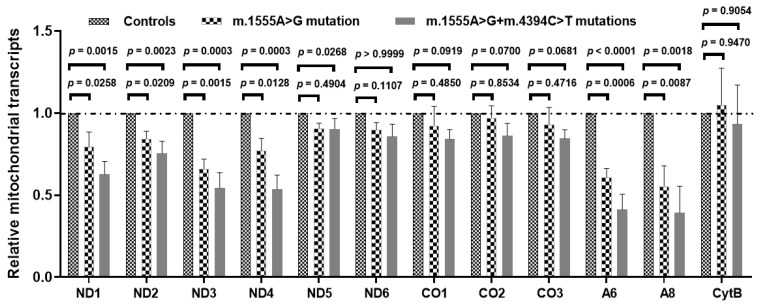
Mt-RNA transcription analysis in three patients with both the m.1555A>G and m.4394C>T mutations, three patients with only the m.1555A>G mutation and three controls.

To explore the synergistic roles of the m.4394C>T mutation on m.1555A>G-induced mitochondrial dysfunction, we evaluated the mt-RNA transcription in patient-derived cell lines and found that significantly lower levels of ND1, ND2, ND3, ND4, A6 and A8 mRNA were present in the cell lines carrying both the m.1555A>G and m.4394C>T mutations when compared with the cells carrying only the m.1555A>G mutation and controls without these primary mutations (Figure 8). This result indicated that the m.4394C>T mutation might, at least partially, block mt-RNA transcription.

### 3.9. Reduced Activities of Complexes I~IV

**Figure 9:**
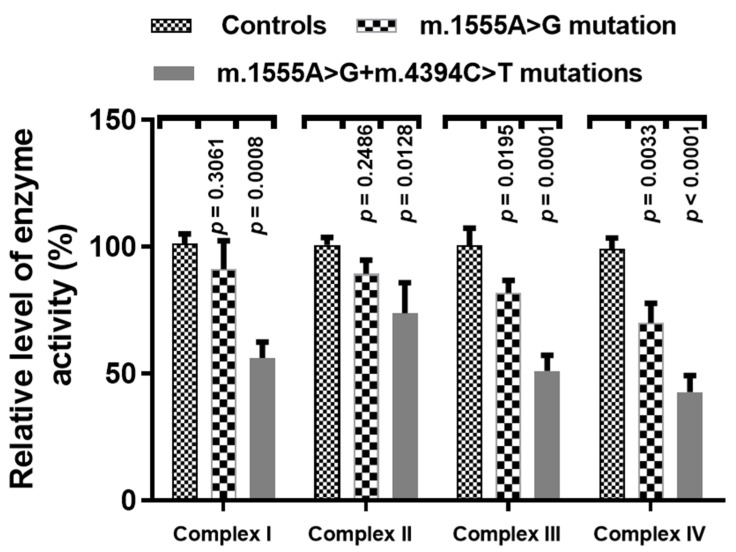
Enzymatic activities of respiratory chain complexes in three patients with both the m.1555A>G and m.4394C>T mutations, three patients with only the m.1555A>G mutation and three controls.

To see the effect of the m.4394C>T mutation on OXPHOS functions, we analyzed the activities of respiratory complexes in three cell lines with both the m.1555A>G and m.4394C>T mutations, three cell lines derived from patients with only the m.1555A>G mutation, and three cells without these mutations. As shown in Figure 9, we noticed that Complexes I~IV were markedly decreased in cells with both mtDNA mutations, as compared with controls (*p* < 0.05 for all).

### 3.10. The m.4394C>T Mutation Increases DNA Damage

**Figure 10:**
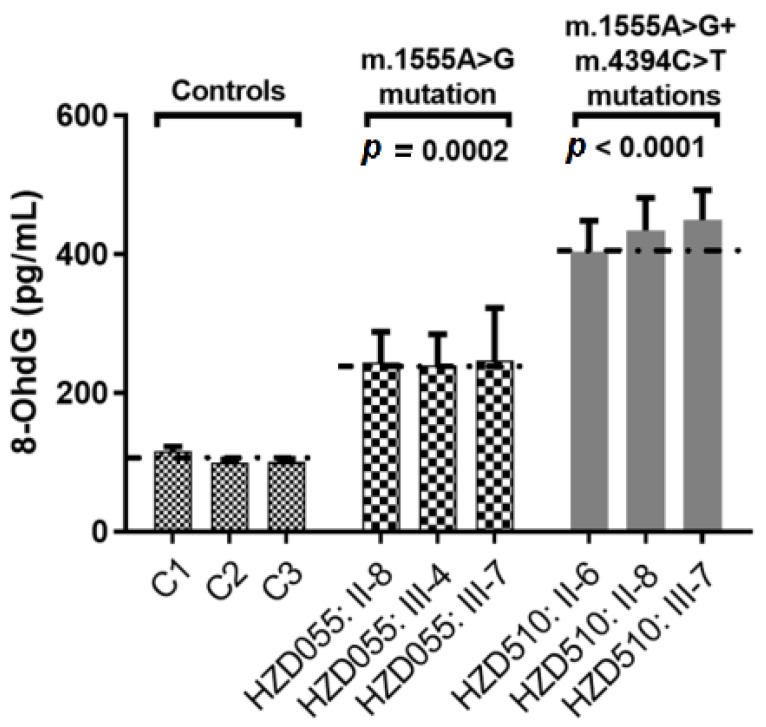
Qualification of serum 8-OHdG levels in three patients with both the m.1555A>G and m.4394C>T mutations, three patients with only the m.1555A>G mutation and three controls.

To see whether the m.4394C>T mutation enhanced the DNA damage, the serum 8-OHdG levels were measured using ELISA. As shown in Figure 10, we found that subjects with both the m.1555A>G and m.4394C>T mutations exhibited higher levels of 8-OHdG than patients with only the m.1555A>G mutation (*p* = 0.0002) and controls without those mutations (*p* < 0.0001).

### 3.11. Mutational Screening for Common Deafness-Associated Nuclear Genes

To uncover the roles of nuclear gene mutations in deafness, we screened for the mutations in common deafness-associated genes (*GJB2*, *GJB3*, *GJB6 SLC26A4* and *TRMU*) in the matrilineal relatives of the HZD055 and HZD510 pedigrees. However, PCR and Sanger sequencing revealed that no functional variants could be identified in these genes, suggesting that nuclear modified genes may not play an active role in m.1555A>G-induced deafness.

### 3.12. Possible Pathogenic Effect of m.4394C>T in AINSHL

As shown in Table 4, according to the pathogenicity scoring system [41], the total score of the m.4394C>T mutation was 8 points, meaning it is “possibly pathogenic” and may cause AINSHL.

## 4. Discussion

The present study has explored the synergistic role of the m.4394C>T mutation in clinical manifestations of deafness-associated m.1555A>G mutation. Hearing loss as a sole clinical phenotype only presented in matrilineal relatives, but not in other members in these pedigrees, suggesting that mitochondrial dysfunctions caused by mtDNA mutations are the molecular basis for this disease. In fact, the m.1555A>G mutation was originally identified in Arab-Israeli families with AmAn-associated hearing loss [48]. Without the usage of AmAn, subjects with the m.1555A>G mutation exhibited a wide range of clinical phenotypes that ranged from mild to severe hearing loss, and even normal hearing, indicating that while the m.1555A>G mutation is a major risk factor for hearing loss, by itself it is not sufficient to produce the phenotype [49], for which it requires other factors such as nuclear genes, AmAn or mtDNA secondary variants [50,51]. In particular, mtDNA haplogroup B4C1C exhibited a higher penetrance and expressivity of m.1555A>G-induced deafness [52]. Recent experimental studies indicate that mitochondrial haplogroup B enhances the risk for hearing impairment in Chinese patients carrying the m.1555A>G mutation [53]. Moreover, mitochondrial haplogroup D4 specific m.5802T>C, m.10454T>C, m.12224C>T and m.11696G>A mutations may enhance m.1555A>G-induced deafness [15]. Sequence characterization of the complete mitochondrial genome of matrilineal relatives from these pedigrees suggested the presence of sets of polymorphisms belonging to mtDNA haplogroup D4g2a [21]. We noticed that the penetrance of hearing loss in HZD055 was 50% including AmAn and 10% excluding AmAn, while in HZD510 it was 30% including AmAn and 0% excluding AmAn. Compared with previous studies, the penetrances of m.1555A>G-induced hearing loss ranged from 22.2% to 73.9% (average: 50.9%, including AmAn), whereas excluding the effects of AmAn, the penetrances varied from 11.1% to 60.8% (average: 30.8%, Table 5) [15,16,20,54]. 

The m.4394C>T mutation resided at position 7 in the 5′ end of tRNA^Gln^, which was well conserved from different vertebrates. Notably, the m.4394C>T mutation introduced a novel 7T-66A base-pairing and was critical for the steady-state level of the tRNA^Gln^. Interestingly, nucleotides at the same position in tRNA^Gly^ (9997T>C) and in tRNA^Ile^ (4269A>G) were regarded as pathogenic mutations for cardiomyopathy [55,56]. Hence, the m.4394C>T mutation may be similar to the m.9997T>C or m.4269A>G mutation, and cause mitochondrial dysfunction that is involved in hearing impairment.

Trans-mitochondrial technology was frequently used to assess the contribution of mtDNA genetic polymorphisms to OXPHOS function since noise from the nuclear genetic background was adjusted. We demonstrated that cybrid cells bearing both the m.1555A>G and m.4394C>T mutations exhibited much more severe effects on mitochondrial dysfunctions than those with only the m.1555A>G mutation and controls. Specifically, we observed drops of approximately 52% and 26% in ATP production in mutant cells carrying both the m.1555A>G and m.4394C>T mutations or mutant cell lines with only the m.1555A>G mutation, respectively, which are below the threshold ATP level for developing a clinical phenotype [57]. Notably, the deficiency of mitochondrial respiratory chain functions will frequently affect MMP, and in this study, approximately 47% and 26% reductions in MMP were found in cell lines carrying both the mtDNA mutations, and in cell lines with only the m.1555A>G mutation, respectively. It had been recognized that loss of MMP may cause cytochrome c to release into the cytoplasm and then induce programmed cell death [58]. Moreover, MMP constitutes an important component of mitochondrial respiration complexes and is associated with many mitochondrial functions, such as ATP formation and mitochondrial protein import [59,60]. In addition, a much lower level in mtDNA content was found in patients with both mutations than in patients with only the m.1555A>G mutation. Indeed, the mtDNA copy number is an important element for mitochondrial replication and transcription, which is critical for cell functions [61]. Alternations in mtDNA copy number are associated with diverse human pathologies [62]. Thus, the abnormal mtDNA copy number and MMP may lead to an increased in ROS production. ROS are the by-products of oxidative stress, and include peroxides, superoxides, hydroxyl radicals and singlet oxygen [63]. It has been documented that ROS plays a crucial role in human physiological and pathophysiological processes [64]. High ROS concentration will react with proteins, lipids, RNA or DNA, and frequently lead to irreversible functional alterations, including regulating mitochondrial functions, apoptosis and signaling transduction [65]. These results indicated that the ATP, MMP, ROS and mtDNA copy number were impaired in cells with both mtDNA mutations.

Next, examination of the RNA level of mtDNA-encoded OXPHOS subunits revealed that the mRNA levels of ND1, ND2, ND3, ND4, A6 and A8 were lower in cybrids carrying both mtDNA mutations than in cybrids with only the m.1555A>G mutation (*p* < 0.05 for all). This result indicates that defective mt-RNA translation caused by both the m.1555A>G and m.4394C>T mutations may be responsible for multiple OXPHOS complex deficiencies in cybrid cells. The altered tRNA^Gln^ metabolism caused by the m.4394C>T mutation led to a defect in mitochondrial translation. In fact, cell lines with both the m.1555A>G and m.4394C>T mutations would perturb the activities of Complexes I–IV and then worsen the defects in mitochondrial respiratory phenotypes associated with the m.1555A>G mutation, as in the case of the m.14692A>G mutation [66]. 

In addition, the level of serum 8-OHdG was much higher in patients with both the m.1555A>G and m.4394C>T mutations, suggesting that the mitochondrial dysfunctions caused by the m.1555A>G mutation may be worsened by the m.4394C>T mutation. Furthermore, the absent of any functional variants in deafness-related common nuclear genes (*GJB2*, *GJB3*, *GJB6,* *SLC26A4* and *TRMU*) in the matrilineal relatives in these pedigrees suggests that nuclear modified genes may not play an active role in deafness expression, hence, the mt-tRNA^Gln^ 4394C>T mutation may enhance the penetrance of m.1555A>G-induced deafness.

## 5. Conclusions

In conclusion, the mt-tRNA^Gln^ 4394C>T mutation may aggravate mitochondrial dysfunction and increase the penetrance of hearing impairment caused by the m.1555A>G mutation.

## Figures and Tables

**Table 1:** Oligonucleotide primers for mtDNA transcription.

Locus	Starting	Ending	Length (bp)	Forward (5′-3′)	Reverse (5′-3′)
**ND1**	3307	4262	956	CCCATGGCCAACCTCCTACTCCTC	AGCCCGTAGGGGCCTACAACG
**ND2**	4470	5511	1042	AACCCTCGTTCCACAGAAGCT	GGATTATGGATGCGGTTGCT
**ND3**	10059	10404	346	ACGAGTGCGGCTTCGACCCT	TCACTCATAGGCCAGACTTAGGGCT
**ND4**	10760	12137	1377	CCCACTCCCTCTTAGCCAATATT	TAGGCCCACCGCTGCTT
**ND5**	12337	14148	1812	TGCTCCGGGTCCATCATC	TGAGTAGTCCTCCTATTTTTCGAATATCT
**ND6**	14149	14673	525	GCCCCCGCACCAATAGGATCCTCCC	CCTGAGGCATGGGGGTCAGGGGT
**CO1**	5904	7445	1542	GCCCCCGATATGGCGTTTCCCCGCA	GGGGTCTCCTCCTCCGGCGGGGTCG
**CO2**	7586	8269	684	ACCAGGCGACCTGCGACTCCT	ACCCCCGGTCGTGTAGCGGT
**CO3**	9207	9990	784	CCCCCAACAGGCATCACCCCGC	ATGCCAGTATCAGGCGGCGGC
**A8**	8366	8572	207	CCCACCATAATTACCCCCATACT	GGTAGGTGGTAGTTTGTGTTTAATATTTTTAG
**A6**	8527	9207	681	TTATGAGCGGGCACAGTGATT	GAAGTGGGCTAGGGCATTTTT
**CytB**	14747	15887	1141	CCCACCCTCACACGATTCTTTA	TTGCTAGGGCTGCAATAATGAA

**Table 2:** Summary of the clinical data of several members in two pedigrees with hearing impairment.

Subject	Sex	Age at Test (Year)	Age at Onset (Year)	Use of AmAn	PTA (Right Ear) (dB)	PTA (Left Ear) (dB)	Level of Hearing Impairment	Audiometric Configuration	Presence of Functional mtDNA Mutations
**HZD055: II-8**	Female	66	31	Gentamycin	105	70	Profound	Slop	m.1555A>G
**HZD055: III-4**	Female	42	20	Kanamycin	21	92	Severe	Slop	m.1555A>G
**HZD055: III-7**	Female	38	19	Gentamycin	106	86	Profound	Slop	m.1555A>G
**HZD510: II-6**	Female	50	45	No	30	30	Mild	Flat	m.1555A>G and m.4394C>T
**HZD510: II-8**	Female	49	22	Gentamycin	54	57	Moderate	Slop	m.1555A>G and m.4394C>T
**HZD510: III-7**	Female	25	24	No	85	71	Severe	Slop	m.1555A>G and m.4394C>T
**HZD055:II-6** **(C1)**	Female	44	/	No	18	20	Normal	Flat	None
**HZD055:III-1 (C2)**	Male	20	/	No	16	18	Normal	Flat	None
**HZD055:III-3 (C3)**	Male	15	/	No	13	17	Normal	Flat	None

Abbreviations: AmAn: aminoglycoside antibiotics; PTA: pure tone audiometry; dB: decibels.

**Table 3:** mtDNA sequence variants in two pedigrees with hearing loss.

Gene	Position	Alternations (Amino Acid)	Conservation (H/B/M/X) ^a^	rCRS ^b^	HZD055	HZD510	Previously Reported ^c^
**D-loop**	73	A→G		A	G	G	Yes
	143	G→A		G	A		Yes
	152	T→C		T		C	Yes
	195	T→C		T	C		Yes
	263	A→G		A	G	G	Yes
	310	T→CTC		T	TC	CTC	Yes
	489	T→C		T	C	C	Yes
	16051	A→G		A		G	Yes
	16129	G→A		G		A	Yes
	16183	A→C		A	C		Yes
	16188	C→T		C	T		Yes
	16223	C→T		C	T	T	Yes
	16311	T→C		T	C	C	Yes
	16362	T→C		T	C		Yes
**12S rRNA**	709	G→A	G/A/A/-	G	A		Yes
	750	A→G	A/A/G/-	A	G	G	Yes
	1438	A→G	A/A/A/G	A	G	G	Yes
	1555	A→G	A/A/A/A	A	G	G	Yes
**16S rRNA**	2706	A→G	A/G/A/A	A	G	G	Yes
	3010	G→A	G/G/A/A	G		A	Yes
	3107	N del		N		N del	Yes
***ND1***	3483	G→A		G		A	Yes
	3970	C→T		C		T	Yes
**tRNA^Gln^**	4394	C→T	C/C/C/C	C		T	Yes
***ND2***	4491	G→A (Val→Ile)	V/I/I/V	G	A		Yes
	4769	A→G		A	G	G	Yes
	4883	C→T		C	T	T	Yes
	4985	G→A		G	A		Yes
	5178	C→A (Leu→Met)	L/T/T/T	C	A	A	Yes
	5231	G→A		G	A		Yes
	5417	G→A		G	A		Yes
***COI***	5978	A→G		A		G	Yes
	6392	T→C		T	C		Yes
	7028	C→T		C	T	T	Yes
***COII***	7976	G→A (Gly→Ser)	G/G/S/G	G		A	Yes
***NC_7***	8271–79	9-bp del		9-bp del			Yes
***A8***	8414	C→T (Leu→Phe)	L/F/M/W	C	T	T	Yes
	8584	G→A (Ala→Thr)	A/V/V/I	G	A		Yes
***A6***	8701	A→G (Thr→Ala)	T/S/L/Q	A	G	G	Yes
	8860	A→G (Thr→Ala)	T/A/A/T	A	G	G	Yes
***CO3***	9428	T→C		T	C		Yes
	9540	T→C		T	C	C	Yes
	9755	G→A		G	A		Yes
	9950	T→C		T		C	Yes
***ND3***	10398	A→G (Thr→Ala)	T/T/T/A	T	G	G	Yes
	10400	C→T		C	T	T	Yes
***ND4***	10873	T→C		T	C	C	Yes
	11050	T→C		T	C		Yes
	11059	C→T		C		T	Yes
	11719	G→A		G	A	A	Yes
	11968	A→T		A	T		Yes
***ND5***	12358	A→G		A	G		Yes
	12705	C→T		C	T	T	Yes
	12711	G→A		G	A		Yes
	12825	T→C		T		C	Yes
***ND6***	14668	C→T		C		T	Yes
***CytB***	14766	C→T (Thr→Ile)	T/S/I/S	C	T	T	Yes
	14783	T→C		T	C	C	Yes
	15043	G→A		G	A	A	Yes
	15301	G→A		G	A	A	Yes
	15326	A→G (Thr→Ala)	T/M/I/I	A	G	G	Yes
	15784	T→C		T		C	Yes

^a^ Conservation of amino acid for polypeptides or nucleotide for rRNAs in human (H), bovine (B), mouse (M), and *Xenopus laevis* (X). ^b^ rCRS: reversed Cambridge References Sequences. ^c^ See the online mitochondrial genome database https://www.mitomap.org/MITOMAP (accessed on 11 September 2022).

**Table 4:** The predicted pathogenicity of the mt-tRNA^Gln^ 4394C>T mutation.

Scoring Criteria	m.4394C>T Mutation	Score/20	Classification
**More than one independent report**	Yes	2	≤6 points: neutral polymorphisms; 7~10 points: possibly pathogenic; 11–13 points (not including evidence from the single-fiber, steady-state level or from trans-mitochondrial cybrid studies): probably pathogenic ≥11 points (including evidence from the single-fiber, steady-state level or from trans-mitochondrial cybrid studies): definitely pathogenic
**Evolutionary conservation of the base pair**	No changes	2	
**Variant heteroplasmy**	No	0	
**Segregation of the mutation with disease**	Yes	2	
**Histochemical evidence of mitochondrial disease**	No evidence	0	
**Biochemical defect in complex I, III or IV**	Yes	2	
**Evidence of mutation segregation with biochemical defect from single-fiber studies**	No	0	
**Mutant mt-tRNA steady-state level or evidence of pathogenicity in trans-mitochondrial cybrid studies**	No evidence	0	
**Maximum score**	Possibly pathogenic	8	

**Table 5:** Summary of clinical and molecular data for 12 families with hearing loss.

Pedigree Name	Number of Matrilineal Relatives	Penetrance of Hearing Loss (Including AmAn) (%)	Penetrance of Hearing Loss (Excluding AmAn) (%)	Primary mtDNA Mutation	Functional mtDNA Mutations	mtDNA Haplogroup	References
**HZD055**	10	30	0	1555A>G	None	D4g2a	This study
**HZD510**	10	50	10	1555A>G	tRNA^Gln^ 4394C>T	D4g2a	This study
**HZD501**	9	22.2	11.1	1555A>G	tRNA^Cys^ 5802T>C	D4b2b	20
**HZD502**	8	37.5	12.5	1555A>G	tRNA^Thr^ 15930G>A	K1a	20
**HZD503**	7	42.8	14.3	1494C>T	tRNA^Lys^ 8343A>G	B4b1c	20
**WZD91**	23	73.9	60.8	1555A>G	tRNA^Ile^ 4317A>G	B4	16
**P206**	7	71.4	28.6	1555A>G	*CO2* 7598G>A	M7b1	54
**WZD11**	19	53	42	1555A>G	*ND4* 11696G>A	D4	15
**WZD12**	12	58	25	1555A>G	CO1/tRNA^Ser(UCN)^ 7444G>A	B4c1	15
**WZD40**	20	30	25	1555A>G	*ND5* 12338T>C	F2	15
**WZD50**	15	53.3	46.7	1555A>G	tRNA^Ser(AGY)^ 12224C>T	D5	15
**WZD32**	12	66.7	41.7	1555A>G	tRNA^Thr^ 15927G>A	B5b1	15

## Data Availability

All the data supporting the results of this study are included in the article.
